# A Medical Student–Delivered Smoking Prevention Program, Education Against Tobacco, for Secondary Schools in Brazil: Study Protocol for a Randomized Trial

**DOI:** 10.2196/resprot.7134

**Published:** 2017-01-30

**Authors:** Luiz Eduardo De Freitas Xavier, Breno Bernardes-Souza, Oscar Campos Lisboa, Werner Seeger, David Alexander Groneberg, Thien-An Tran, Fabian Norbert Fries, Paulo César Rodrigues Pinto Corrêa, Titus Josef Brinker

**Affiliations:** ^1^ School of Medicine Federal University of Ouro Preto Ouro Preto Brazil; ^2^ Universities of Giessen and Marburg Lung Center, Member of the German Center for Lung Research Department of Internal Medicine University of Giessen Giessen Germany; ^3^ Institute of Occupational Medicine, Social Medicine and Environmental Medicine Goethe University Frankfurt am Main Germany; ^4^ Pulmonary and Respiratory Critical Care Medicine Thoraxklinik and Translational Lung Research Center Heidelberg, Member of the German Center for Lung Research University of Heidelberg Heidelberg Germany; ^5^ Saarland University Medical Center and Saarland University Faculty of Medicine Saarland University Homburg Germany

**Keywords:** photoaging, schools, tobacco prevention, adolescents, medical students

## Abstract

**Background:**

Smoking is the largest preventable cause of morbidity and mortality in Brazil. Education Against Tobacco (EAT) is a large network of medical students in 13 countries who volunteer for school-based prevention in the classroom setting. A recent quasi-experimental EAT study conducted in Germany showed significant short-term smoking cessation effects on 11- to 15-year-old adolescents.

**Objective:**

The aim of this study is both to describe and to provide the first randomized long-term evaluation of the EAT intervention involving a photoaging app for its effectiveness to reduce the smoking prevalence among 12- to 17-year-old pupils in Brazilian public schools.

**Methods:**

A randomized controlled trial will be conducted among approximately 1500 adolescents aged 12 to 17 years in grades 7-11 of public secondary schools in Brazil. The prospective experimental study design includes measurements at baseline and at 6 and 12 months postintervention. The study groups will consist of randomized classes receiving the standardized EAT intervention (90 minutes of mentoring in a classroom setting) and control classes within the same schools (no intervention). The questionnaire measures smoking status, gender, social, and cultural aspects as well as predictors of smoking. Biochemical validation of smoking status is conducted via random carbon monoxide measurements. The primary end point is the difference of the change in smoking prevalence in the intervention group versus the difference in the control group at 12 months of follow-up. The differences in smoking behavior (smoking onset, quitting) between the 2 groups as well as effects on the different genders will be studied as secondary outcomes.

**Results:**

The recruitment of schools, participating adolescents, and medical students was conducted from August 2016 until January 2017. The planned period of data collection is February 2017 until June 2018. Data analysis will follow in July 2018 and data presentation/publication will follow shortly thereafter.

**Conclusions:**

This is the first evaluative study of a medical student–delivered tobacco prevention program in Brazil and the first randomized trial on the long-term effectiveness of a school-based medical student–delivered tobacco prevention program in general.

**ClinicalTrial:**

ClinicalTrials.gov NCT02725021; https://clinicaltrials.gov/ct2/show/NCT02725021 (archived by WebCite at http://www.webcitation.org/6njy3nNml)

## Introduction

Smoking is one of the main risk factors both for mortality and disability-adjusted life years (DALYs) for noncommunicable chronic diseases in Brazil and globally [1-3]. A study published in 2011 estimated the tobacco-related burden in Brazil and found that smoking was accountable in that year for 147,072 deaths and 2.69 million DALYs, representing a direct cost for the health system of $23.37 billion Brazilian real (US $7.37 billion) [4].

According to a large national study that was conducted in 2012 with 61,037 9th graders in Brazil, over 30.0% of 13- to 15-year-olds try smoking before the age of 12 (National School Health Survey—PeNSE) [5]. The newest data from the 2016 ERICA study with 74,589 Brazilian 12- to 17-year-old participants revealed a current smoking prevalence of 5.7% in this young age group with more smokers in public than in private schools (5.9% vs 4.4%) [6]. Although smoking rates among adolescents and emerging adults have substantially declined since the early 2000s, prevalence is still high and strong socioeconomic and educational inequalities in smoking exist in Brazil [6].

Founded in 2012 in Germany, Education Against Tobacco (EAT) has now enrolled participants in over 80 medical schools in 13 countries worldwide. The network has its roots in the school-based interventions delivered by medical students that cost about US $20 per participating class and reach up to 40,000 students per year worldwide. However, the network is also involved in medical education research on smoking cessation counseling, science-based multilanguage apps, and public awareness and advocacy for tobacco control [7-9].

The school-based intervention has only been evaluated for short-term effects with a quasi-experimental design with multiple potential sources of bias [10,11]. However, its preliminary evaluation involving 1474 students showed a significant reduction in the smoking prevalence of secondary school students in Germany at 6 months of follow-up after interventions motivating them to quit [10,11]. After this first evaluation, the curriculum was optimized for students with a lower educational level because the intervention was less effective in this subgroup [11]. This was done by making the program more interactive and by developing and involving a photoaging app (Smokerface) into our classroom intervention because published research suggests that there is effectiveness of photoaging strategies for our age group [7].

The European Smoking Prevention Framework Approach (ESFA) program developed and tested a smoking prevention program for adolescents aged between 12 and 15 years (students enrolled in the 7th-9th school grades) in 6 European countries [12]. Studies on the ESFA project have shown mixed outcomes: only Portugal, Spain, and Finland achieved positive results [13]. We suspect there will be differences between the outcomes in Brazil and the ones we found in Germany.

Smoking is largely conceptualized to medical students as an adult health problem, although most long-term smokers started as adolescents. Recent studies indicate that tobacco addiction is substantially undertreated by physicians in comparison with other chronic conditions such as diabetes or hypertension [14-16]. The authors conclude that this is mainly due to lack of motivation, skills, and knowledge from the medical community [14-16]. There is evidence that medical student training addressing adolescent health promotion to prevent smoking can increase the frequency of obtaining smoking status of and providing advice to patients [17]. The importance of primary and secondary prevention of smoking uptake has led to calls for a greater engagement and training of future physicians in tobacco control [14]. In this context, the EAT network not only provides school-based prevention but also sensitizes future physicians to the importance of tobacco prevention and cessation [10,11,18].

The aim of this publication is both to describe the optimized EAT intervention and to present the study protocol of a trial designed to determine the long-term effectiveness of the school-based EAT intervention in reducing the smoking prevalence among secondary school students in Brazil.

## Methods

### Ethics Approval and Consent to Participate

In accordance with Good Epidemiologic Practice guidelines [19], the study protocol was submitted for approval by the responsible ethics committee (Federal University of Ouro Preto, Brazil), and consent was obtained. All legal and data protection issues were discussed with the responsible authorities, and all participants are required to provide active informed consent.

### The Education Against Tobacco Intervention

#### Overview

The school-based intervention under evaluation consists of a 90-minute module in the classroom setting. It is presented by 2 medical students per classroom to about 25 pupils at a time discussing features of smoking that students can relate to in their everyday life in a gain-framed and interactive manner. The goal is to initiate and guide the student evaluation process of smoking with age-appropriate information that helps them to reframe a positive nonsmoking image by asking for their opinions and views in small group settings. The students form 4 groups (mostly with their friends) and rotate to 4 different stations in the classroom.

#### Station 1

This station, built up near a window or outside, discusses different tobacco products and extraction of substances of tobacco smoke. In the first part, different products (including e-cigarettes, a water pipe, and cigarettes) are displayed and their functionality and harmfulness are discussed in a gain-framed manner.

Second, the students are instructed to conduct an experiment using a hand-vacuumizer, a napkin, a plastic bottle and a cigarette. The napkin is put between cigarette and bottle and burned down to demonstrate the ingredients of the smoke by the discoloration of the napkin. The process is repeated with e-cigarettes that show little discoloration and are regarded as less harmful but still contain cancerogenic substances [20].

#### Station 2

The attractiveness and photoaging consequences of nonsmoking and mechanisms related to the face are discussed. In the first part, pictures of monozygotic non-/smoking twins are displayed that were extracted from the publication of Okada et al [21]. The students are asked which twin is the smoker and what differences they note.

**Figure 1:**
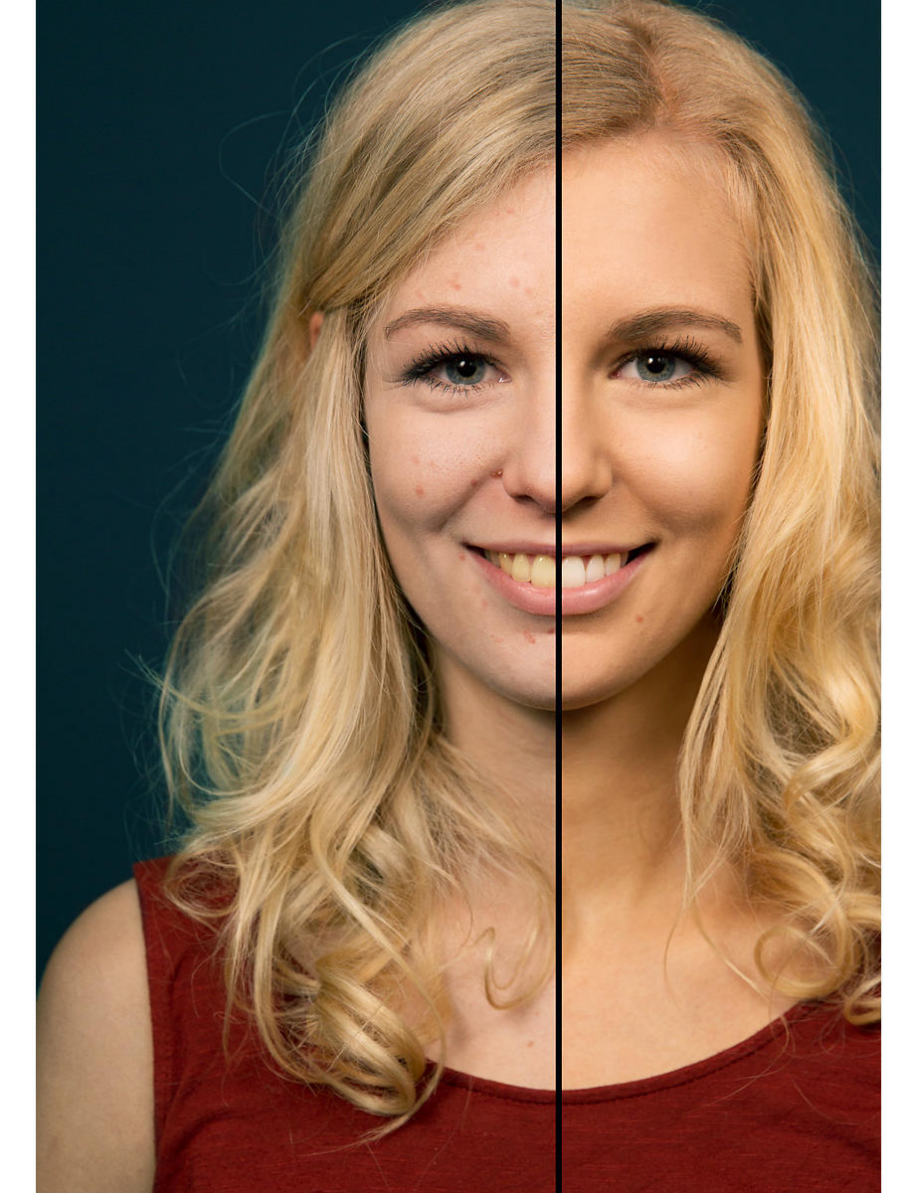
Photoaged image of a 17-year-old woman showing the consequences of smoking 1 pack a day for 1 year (vs nonsmoking).

**Figure 2:**
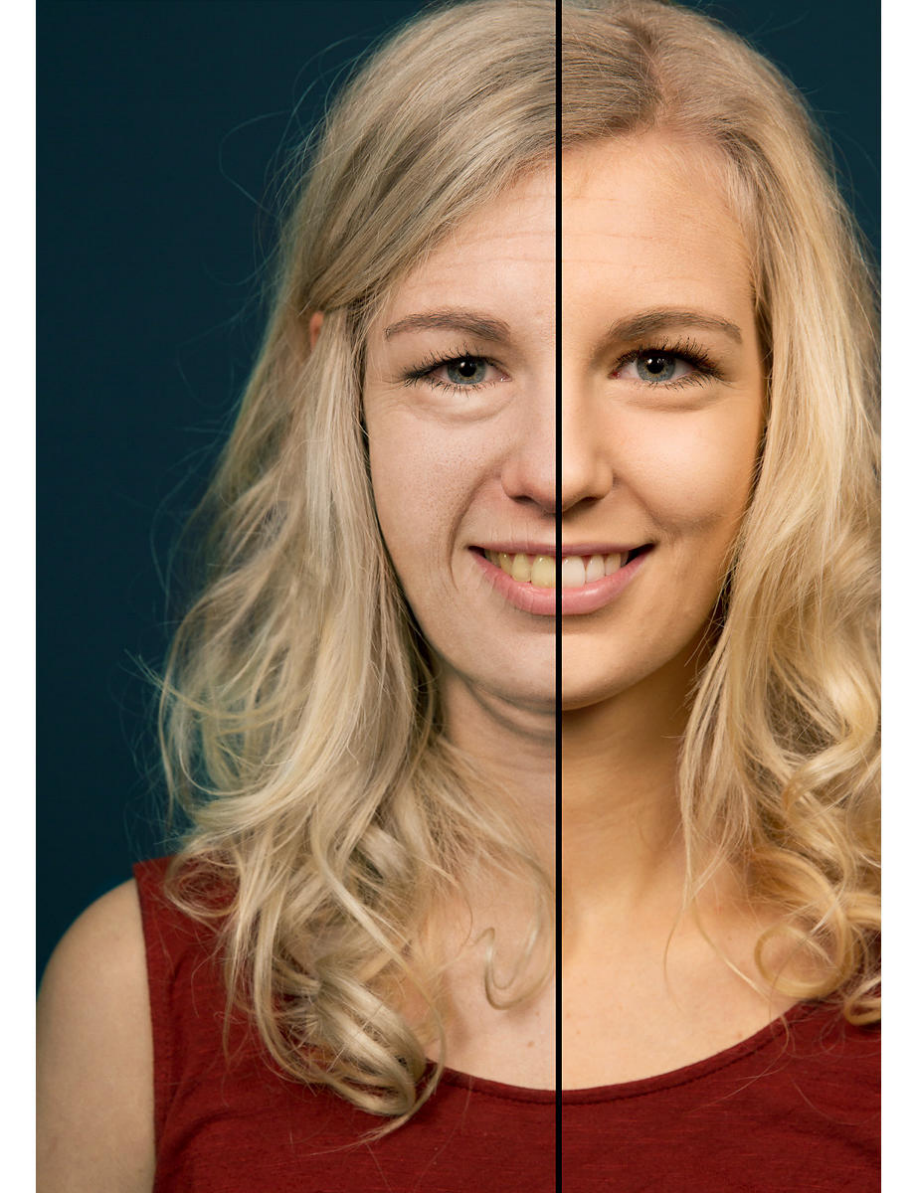
Photoaged image of a 17-year-old woman showing the consequences of smoking 1 pack a day for 15 years (vs nonsmoking).

In the second part, Galaxy Tab E tablets (Samsung Electronics Inc, Seoul, Korea) are used to show each student the effects of non-/smoking on their own faces by the help of the photoaging app Smokerface that we described and piloted in great detail elsewhere [7,9]. By this means, the students’ faces are captured via a selfie and photoaged into 1- and 15-years-older versions of themselves (normal aging vs normal aging plus smoking) (Figures 1 and 2 and Multimedia Appendix 1 demonstrate the effects of the app). The app’s underlying aging algorithms are based on publications showing an increased risk for acne, pale skin, wrinkles, and other capillary and connective tissue changes in smokers. The use of the app influences numerous predictors of smoking in students of this age group in accordance with the theory of planned behavior and as demonstrated in our recent paper [9].

#### Station 3

Performance benefits of nonsmoking (physical performance, stress, common colds) and understanding the mechanisms of how tobacco smoking affects the body with age-appropriate examples (eg, occluded vessels lead to loss of connective tissue in women’s breasts which equals less volume and tightness and impotence in both men and women [22], pale skin, mechanisms of acne); this is explained via pencil-and-paper drafts and interactive questions. In addition, obesity [23,24], lung growth [25], and body growth impairment in adolescent smokers are discussed on a body model with pencil-and-paper sketches and by the use of growth curves [24].

#### Station 4

The aim of this station is to discuss the student’s own experiences with tobacco and how they reacted in the past to peer pressure and to the strategies of the tobacco industry to influence their decision. The groups’ knowledge and experience is shared and discussed in a team setting where the medical students take the role of older friends by complementing the students’ experiences with their own experiences to increase the perceived self-efficacy of the students, which is the most important predictor of future smoking in accordance to the theory of planned behavior [26]. It has been shown to predict both the intention to smoke and actual smoking behavior in a meta-analysis [27].

At the end of the classroom seminar we ask for the students' final judgments on smoking to create positive peer pressure and influence the students’ subjective norm in accordance with the theory of planned behavior [26]. As a final exercise, all students breathe through a straw after having physically exercised in the classroom together to learn how lung impairment due to smoking feels with exercising.

Overall, long-term health consequences are not discussed in great detail as fear approaches were proven to be ineffective and information on the diseases can be found on every cigarette pack [28]; besides that, these long-term harms (such as lung cancer, vascular disease, and chronic pulmonary disease) are too far in the future to fathom. Instead, the program focuses on information relevant for teenagers, particularly related to attractiveness and physical performance, as both of these aspects have been shown in several publications to strongly influence the behavior, self-confidence, and quality of life of adolescents [29-31].

### Design

**Figure 3:**
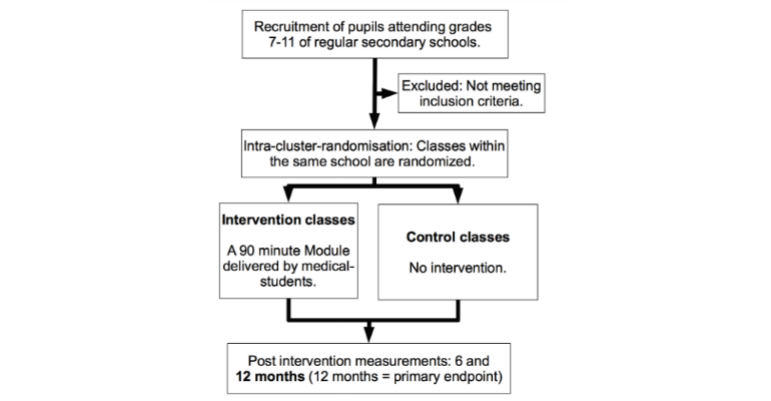
Study design.

A randomized controlled trial will be conducted among approximately 1500 adolescents in grades 7-11 of secondary schools in Brazil (Figure 3). The planned study period is February 2017 until June 2018. The prospective experimental study design includes measurements at baseline (t1) and 6 months (t2) and 12 months (t3) postintervention. The study groups will consist of randomized classes receiving the standardized EAT intervention (90 minutes of mentoring in a classroom setting) and control classes within the same schools (no intervention). The study questionnaire that measures smoking status and gender, social, and cultural aspects uses items from previously tested and published questionnaires. By the use of the conceptual method for translation described by the World Health Organization/United Nations Economic and Social Commission for Asia and the Pacific Project on Health and Disability Statistics, the items were translated to Portuguese [32] and afterwards pretested with students from Brazil [32]. Smoking status is validated by a random carbon monoxide (CO) breathing test at baseline and endline.

Baseline collection will be completed by mid-February 2017. The intervention will be conducted from end of February 2017 until May 2017. The first follow-up survey will be 6 months thereafter. At 1-year follow-up (May/June 2018), the data collection is complete.

### Randomization

In accordance with the guidelines of Good Epidemiologic Practice, randomization is externally and centrally performed via computer by a statistician from the University of Gießen, Germany, on the class level within each school (group allocation is 1:1), which is the strongest method according to the recent Cochrane Analysis [19,33]. Different educational levels within 1 school grade are used for stratification.

### Participants and Sample Size

In accordance with the first evaluation of our program in Germany, a total of 1500 eligible secondary school students will be recruited [7]. Inclusion criteria are the attendance of grades 7-11 at public secondary schools in Brazil with age ranging from 12 to 17 years. Exclusion criteria are defined as inability to fulfill the inclusion criteria (ie, private schools, different grades). The age range for inclusion in the study is higher than in our German study [10] as the median age of smoking onset is higher in Brazil than in Germany [6,11]. As we expect the same loss-to-follow-up effect as in our quasi-experimental trial in Germany (18.6%), a test power of 70% and a 2-sided alpha of .05 are considered appropriate for our primary end point.

### Data Collection

A pencil-and-paper questionnaire is used for the collection of the data and the smoking status is biochemically validated by randomized CO measurements in the exhaled air of the students. In addition to sociodemographic data (age, gender, and school type), the questionnaire will capture the smoking status of the school students concerning cigarettes and multiple other tobacco products (such as e-cigarettes and water pipes). These items are based on 3 established studies [34-36] and were either used in the original form or adapted to the specific circumstances of this study.

The class teachers supervise their classes during the completion of the questionnaire and seal them right after completion for confidentiality reasons. The envelopes are then shipped to the Federal University of Ouro Preto in Brazil where they are opened and the data entry is performed.

### Biochemical Validation

Using a portable CO analyzer (Smokerlyzer piCO+, Bedfont Scientific Ltd), CO testing will be randomly performed at baseline and endline in at least 10% of students [37]. CO measurements will be supervised by medical students with recordings made in the afternoon. The cutoff point is defined as 6 parts per million or less for nonsmoking and more than 6 parts per million for smoking [38-40].

### Outcomes

The primary end point is the difference of the change in smoking prevalence in the intervention group versus the difference in the control group at 12 months of follow-up. The same longitudinal prevalence effects are studied on our females exclusively as a secondary outcome because our photoaging intervention is suspected to be more successful in females [41]. As our sample size is quite small, the secondary outcome criteria is met if 20% fewer females start smoking in the intervention group during the study period of 1 year. The differences in smoking behavior (smoking onset, quitting) between the 2 groups will be studied as additional secondary outcomes with a positive 20% difference in change of behavior set as met outcome criteria. A smoker is defined as a pupil who claims to smoke at least once in the past 30 days within the survey.

### Data Entry

The data entry is performed manually at the Federal University of Ouro Preto in Brazil by the help of the newest version of Excel (Microsoft Corp) spreadsheet.

### Analysis

To examine baseline differences we will use chi-square tests (categorical variables) and *t* tests (continuous variables). The effects of predictors (gender, culture, and social characteristics) on smoking behavior after 12 months (t3) will be calculated by robust panel logistic regression analysis. The significance level is 5% for *t* tests (double-sided) and 95% for confidence intervals (double-sided). Statistical analysis (intention-to-treat) will be performed using SPSS Statistics version 23 (IBM Corp) and STATA 14 (StataCorp LLC). In our sample, the group allocation is not on the individual level but on the class level. In order to take into account this clustering statistically we will use robust panel logistic regression (xtlogit procedure with vce [cluster] option). This procedure is also used to calculate the difference from t1 to t3 of the smoking prevalence in the control group versus the difference from t1 to t3 in the intervention group (our primary end point) by the help of STATA 14.

## Results

TJB raised a grant from the German Heart Foundation that provided funding for the Smokerface App and a grant from the German Center for Lung Research that funded the CO measurement device and the Samsung tablets. The Federal University of Ouro Preto will contribute to the project providing logistic support and copies of the questionnaire to be distributed to every participating student.

The recruitment of schools, participating adolescents, and medical students began in August 2016 and was ongoing until the end of January 2017. The planned period of data collection is February 2017 until June 2018. Data analysis will follow in July 2018 and data presentation/publication will follow shortly thereafter.

## Discussion

### Summary

Tobacco prevention programs for secondary schools conducted by physicians have shown short-term and long-term effectiveness [42-44]. EAT is a novel, fast-growing network of volunteering medical students supported and implemented by medical schools worldwide which has never been evaluated for long-term effects in form of a randomized trial. At the same time, it is valuable to sensitize future physicians while they are still in medical school to tobacco control and to develop their skills, highlighting the associated responsibilities within communities, as they are not only in charge of educating and assisting their future patients effectively about cessation measures but also to develop advocacy skills [45,46]. For instance, at 9 medical schools in Germany, novel smoking cessation counseling teaching courses for medical students have been established by medical students involved in the EAT network. However, the school-based aspect of this network appears to be the most attractive one for medical students not sensitized for the topic as a whole yet.

An important gap in the literature that is addressed by EAT is the fact that there are no guidelines regarding what school-based tobacco prevention programs must specifically cover [47]. The field of school-based tobacco prevention awaits age-appropriate and culturally innovative interventions that can be deemed as effective and then serve as a model to be reproduced. Our study has the potential to directly contribute to tobacco control programs worldwide as our detailed intervention and specific mentoring and training on how to do so are made freely available through the EAT network. The key point in the novelty of the EAT intervention is the use of communication and strategies that are interactive and in line with information relevant for adolescents, especially the smoking-related effects on appearance and physical performance.

### Limitations

Limitations and advantages of the CO testing in exhaled air have been described in great detail elsewhere [37]. Conclusively, our method used for biochemical validation is a widely accepted cost-effective procedure even if it does not have the sensitivity of cotinine saliva testing, which is far more costly [33].

As our research is not conducted multinationally, we can not generalize its results to all cultural backgrounds. However, our intervention involves a photoaging intervention and outward appearance has been described to be the most relevant component of the self-concept in adolescents from multiple cultural backgrounds [48], which increases the international validity of our research.

### Conclusions

This is the first evaluative study of a medical student–delivered tobacco prevention program in Brazil and the first randomized trial on the effectiveness of school-based medical student–delivered tobacco prevention in general. Our research has the potential to foster the way for EAT in Brazil by measuring its effects on different gender, social, and cultural backgrounds. Health systems worldwide could benefit from the development of such novel and low-cost school-based tobacco prevention programs.

## Appendix

Multimedia Appendix 1 3-Dimensional animation of a selfie via the Smokerface app illustrating one of the touch effects (cough) the students can trigger on their own selfies.

## Abbreviations

CO: carbon monoxide
DALY: disability-adjusted life year
EAT: Education Against Tobacco
ESFA: European Smoking Prevention Framework
